# Interpretable clinical visualization model for prediction of prognosis in osteosarcoma: a large cohort data study

**DOI:** 10.3389/fonc.2022.945362

**Published:** 2022-08-02

**Authors:** Wenle Li, Genyang Jin, Huitao Wu, Rilige Wu, Chan Xu, Bing Wang, Qiang Liu, Zhaohui Hu, Haosheng Wang, Shengtao Dong, Zhi-Ri Tang, Haiwen Peng, Wei Zhao, Chengliang Yin

**Affiliations:** ^1^ Department of Orthopaedic Surgery, People's Hospital of Xinjiang Uygur Autonomous Region, Urumqi, Xianyang, China; ^2^ Department of Orthopedics, Hospital of People's Liberation Army of China (PLA), Wuxi, China; ^3^ Intelligent Healthcare Team, Baidu Inc., Beijing, China; ^4^ College of Information and Electrical Engineering, China Agricultural University, Beijing, China; ^5^ Clinical Medical Research Center, Xianyang Central Hospital, Xianyang, China; ^6^ Department of Spine Surgery, Liuzhou People's Hospital, Liuzhou, China; ^7^ Department of Orthopaedics, The Second Hospital of Jilin University, Changchun, China; ^8^ Department of Spine Surgery, Second Affiliated Hospital of Dalian Medical University, Dalian, China; ^9^ School of Physics and Technology, Wuhan University, Wuhan, China; ^10^ Orthopaedic Department, The Fourth Medical Center of People's Liberation Army of China (PLA) General Hospital, Beijing, China; ^11^ Faculty of Medicine, Macau University of Science and Technology, Macao, China

**Keywords:** osteosarcoma, SEER, multicenter study, nomogram, web calculator, prediction model

## Abstract

**Background:**

Currently, the clinical prediction model for patients with osteosarcoma was almost developed from single-center data, lacking external validation. Due to their low reliability and low predictive power, there were few clinical applications. Our study aimed to set up a clinical prediction model with stronger predictive ability, credibility, and clinical application value for osteosarcoma.

**Methods:**

Clinical information related to osteosarcoma patients from 2010 to 2016 was collected in the SEER database and four different Chinese medical centers. Factors were screened using three models (full subset regression, univariate Cox, and LASSO) *via* minimum AIC and maximum AUC values in the SEER database. The model was selected by the strongest predictive power and visualized by three statistical methods: nomogram, web calculator, and decision tree. The model was further externally validated and evaluated for its clinical utility in data from four medical centers.

**Results:**

Eight predicting factors, namely, age, grade, laterality, stage M, surgery, bone metastases, lung metastases, and tumor size, were selected from the model based on the minimum AIC and maximum AUC value. The internal and external validation results showed that the model possessed good consistency. ROC curves revealed good predictive ability (AUC > 0.8 in both internal and external validation). The DCA results demonstrated that the model had an excellent clinical predicted utility in 3 years and 5 years for North American and Chinese patients.

**Conclusions:**

The clinical prediction model was built and visualized in this study, including a nomogram and a web calculator (https://dr-lee.shinyapps.io/osteosarcoma/), which indicated very good consistency, predictive power, and clinical application value.

## Background

Osteosarcoma, the most frequent primary malignancy of bone, accounting for approximately 35% of bone malignancy (1), originates from malignant mesenchymal cells (2), which produce osteoid and/or immature bone (3). Surgery combined with peri-operative chemotherapy is the current treatment while local therapy alone is insufficient (4). The presence or absence of metastases has become an important prognostic factor. Studies have shown that the 5-year survival rate for primary focus without metastases is more than 65% (5–7). Certain variables cannot explain the complicated survival rate. For diagnosis and treatment option, the American Joint Committee on Cancer (AJCC) system (8) and Enneking system (9) are popular. Factors of these systems can imply survival duration with treatment option roughly, but it is limited. A prediction model for survival is urgent for further prognosis prediction and instructive therapy selection (10, 11).

Osteosarcoma incidence remains low relative to other tumors (12). Therefore, a sufficient number of subjects are quite challenging. The Surveillance, Epidemiology, and End Results (SEER) database is an authoritative cancer statistics database in the United States that records morbidity, mortality, and incidence information for millions of patients with malignancies. Currently, although there have been relevant studies on osteosarcoma based on the SEER database, these prediction models showed a lower power (almost AUC < 0.8) or have no external data validation (13–15).

In this study, we built models based on osteosarcoma patients’ data in the SEER database using three models, and the apt model was visualized. The validation data set from four different regional medical centers in China presented great power and credibility of the apt model. The nomogram and the web calculator were visualized, possessing good consistency and clinical application value.

## Methods

### Clinical information and selection criteria

SEER*STAT (version 8.3.5) software was used to extract data including patient demographic characteristics, clinicopathological treatment, and patient treatment (surgery, radiotherapy, and chemotherapy) information.

SEER data inclusion criteria were as follows: (1) primary malignant tumor of osteosarcoma with International Classification of Diseases of Oncology ICD-O codes 9180, 9181, 9182, 9183, 9184, 9185, 9186, 9187, 9192, 9193, 9194, and 9200; (2) SEER database after 2010 incorporated relevant metastatic site information and included patients diagnosed between 2010 and 2016; (3) osteosarcoma was the first and only primary malignancy; (4) complete clinical information, including age at diagnosis, sex, race, primary site, tumor size, tumor stage and grade, metastatic site, surgery, and whether radiotherapy and chemotherapy were administered; (5) diagnosis was from surviving patients and did not include cadavers; (6) complete follow-up information was available; and (7) known cause of death and survival time after diagnosis.

The multicenter data were obtained from four medical institutions in China: the Second Affiliated Hospital of Jilin University, the Second Affiliated Hospital of Dalian Medical University, Liuzhou People’s Hospital, and Xianyang Central Hospital. The follow-up period was more than 3 years. Three investigators were responsible for data acquisition at each institution during the survey period. Tumor size and stage were provided by the surgeon, and pathological grading was diagnosed by a senior pathologist at each hospital, or in case of uncertainty, confirmed by a pathologist at the Second Affiliated Hospital of Jilin University. Data were extracted by two of the three investigators, and data check was performed by the third one. All data were checked for consistency and date was sorted using Microsoft Excel (Microsoft Excel, 2013, Redmond, USA).

Exclusion criteria were as follows: (1) incomplete clinicopathological and survival information; (2) unknown tumor size, stage, and race; and (3) vacant data.

### Calibration of prediction model parameters and data baseline

Considering the characteristics of the SEER database and the multicenter study, we tried to unify the data standard. Three categories of race in SEER data were white, black, and other without specific subdivisions, while the race of real multicenter data from China was classified to “other”. Treatment modality included surgery, chemotherapy, and radiotherapy, but the SEER database did not record treatment details; thus, it could only be classified as No (treatment) or Yes (treatment). Some patients were coded “999” on tumor size in the SEER database, which meant that their tumor size could not be assessed. To minimize data bias, we used x-tile to find the cutoff value of the data that can assess the tumor size, converting the tumor size from a continuous variable to a categorical variable.

Baseline tables were drawn for the modeling and validation group data, independent samples *t*-tests were used for continuous variables, and chi-square tests were used for categorical variables. Heat maps were plotted to show the frequencies and correlations between the parameters.

### Selection of the prediction model

Three methods were used to screen variables in this study: (1) univariate Cox with *p* < 0.05 as a cutoff for screening variables and forest plot; (2) full subset regression to adjust for *R*² maxima to determine the best combination of variables; and (3) the LASSO regression and cross-validation to determine the combination of variables by the *λ* value while the mean squared error (MSE) was minimal.

The variables of the three methods were screened by using stepwise backward regression to achieve the minimum value of Akaike’s Information Criterion (AIC). The models constructed by the three methods were compared by receiver operating characteristic (ROC) curves, and that with the largest area under the curve (AUC) was selected as the final model.

### Survival analysis

In the prognostic analysis, Kaplan–Meier was used to estimate survival curves for each variable, and a log-rank test was used to determine the significant difference. Multivariate Cox regression analysis was used, and forest plots were drawn.

### Development and visualization of prediction models

A nomogram was constructed using the parameters screened from the multivariate Cox results. For application convenience, a user-friendly web calculator was provided. Meanwhile, we built the decision tree.

### Model validation and clinical application assessment

The actual and predicted probabilities were compared using calibration curves for the training and validation sets to evaluate the model consistency. The ROC of the validation set was plotted, and AUC was calculated to evaluate the prediction accuracy of the prediction model. Decision curve analysis (DCA) was used to evaluate the clinical application value.

### Statistical analysis

Cutoff values were obtained by x-title software. Statistical methods and plotting, including *t*-test, chi-square test, LASSO, full subset regression, heat map, Kaplan–Meier, forest plot, nomogram, ROC curve, calibration plot, and DCA curve, were performed by R version 4.0.5. *p* < 0.05 was considered statistical significance.

## Results

### Continuous variables transformed into categorical variables

**Figure 1:**
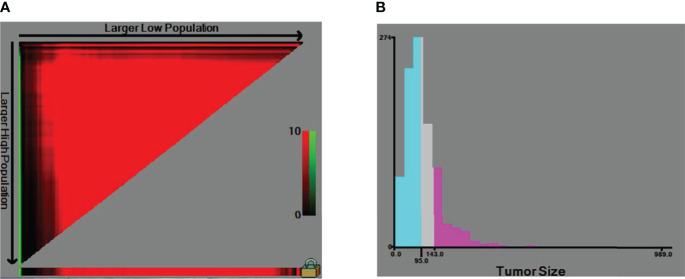
The cut-off of tumor size. **(A)** The x-tile software calculated the optimal division of tumor size. **(B)** Categorical variables.

In **Figure 1**, the x-tile software calculated the optimal division of tumor size into the following groups: less than or equal to 95 mm, 95–127 mm, and more than 127 mm. Therefore, the continuous variable in tumor size was transformed into categorical variables in the three groups of ≤95, 95–127, and >127. Other patients coded “999” on tumor size were allocated to “Unable to evaluate”.

### Baseline data about SEER and multicenter data

Based on the SEER database, information was collected on all patients with osteosarcoma between 2010 and 2016, and according to inclusion and exclusion criteria, 1,144 patients were finally included, while a total of 112 patients were included in the Chinese multicenter data. Flowchart of data collection and analysis was shown in **Figure S1**.

**Table 1** showed the demographic, clinicopathological, and treatment data characteristics of the SEER database versus the Chinese multicenter. Among the statistically significant differences between the two cohorts were race and chemotherapy. In the SEER data, Caucasians predominated, followed by blacks and other ethnicities. The multicenter data had a only Chinese population and a higher proportion of chemotherapy for osteosarcoma in China. No significant differences existed regarding other characteristics between the two groups (**Table 1**).

**Table 1:** Baseline data table of the training group and the validation group.

Variable	Level	SEER data (training group *N* = 1,144)	Multicenter data (validation group *N* = 112)	*p*
Age, mean (SD)	NA	33.47 (24.26)	31.62 (24.88)	0.443
Survival times, mean (SD)	NA	29.91 (22.54)	30.10 (24.24)	0.933
Race (%)	Black	168 (14.7)	0 (0.0)	<0.001
	Other	116 (10.1)	112 (100.0)	
	White	860 (75.2)	0 (0.0)	
Primary site (%)	Axis bone	309 (27.0)	27 (24.1)	0.349
	Limb bone	738 (64.5)	79 (70.5)	
	Other	97 (8.5)	6 (5.4)	
Year of diagnosis (%)	2010	180 (15.7)	18 (16.1)	0.001
	2011	178 (15.6)	20 (17.9)	
	2012	197 (17.2)	16 (14.3)	
	2013	169 (14.8)	17 (15.2)	
	2014	193 (16.9)	14 (12.5)	
	2015	198 (17.3)	15 (13.4)	
	2016	29 (2.5)	12 (10.7)	
Laterality (%)	left	494 (43.2)	43 (38.4)	0.08
	Not a paired site	163 (14.2)	10 (8.9)	
	right	487 (42.6)	59 (52.7)	
Stage group (%)	I	182 (15.9)	17 (15.2)	0.816
	II	521 (45.5)	49 (43.8)	
	III	44 (3.8)	7 (6.2)	
	IV	263 (23.0)	26 (23.2)	
	UNK stage	134 (11.7)	13 (11.6)	
T (%)	T1	388 (33.9)	38 (33.9)	0.294
	T2	523 (45.7)	46 (41.1)	
	T3	35 (3.1)	7 (6.2)	
	TX	198 (17.3)	21 (18.8)	
N (%)	N0	1,011 (88.4)	93 (83.0)	0.255
	N1	35 (3.1)	5 (4.5)	
	NX	98 (8.6)	14 (12.5)	
M (%)	M0	892 (78.0)	84 (75.0)	0.547
	M1	252 (22.0)	28 (25.0)	
Radiation (%)	No	999 (87.3)	104 (92.9)	0.119
	Yes	145 (12.7)	8 (7.1)	
Chemotherapy (%)	No	260 (22.7)	14 (12.5)	0.017
	Yes	884 (77.3)	98 (87.5)	
Tumor size (%)	>127	167 (14.6)	22 (19.6)	0.349
	≤95	552 (48.3)	47 (42.0)	
	59–127	235 (20.5)	21 (18.8)	
	Unable to evaluate	190 (16.6)	22 (19.6)	
Bone metastases (%)	No	1,044 (91.3)	102 (91.1)	0.981
	Unknown	47 (4.1)	5 (4.5)	
	Yes	53 (4.6)	5 (4.5)	
Surgery (%)	No	230 (20.1)	24 (21.4)	0.834
	Yes	914 (79.9)	88 (78.6)	

**Figure 2:**
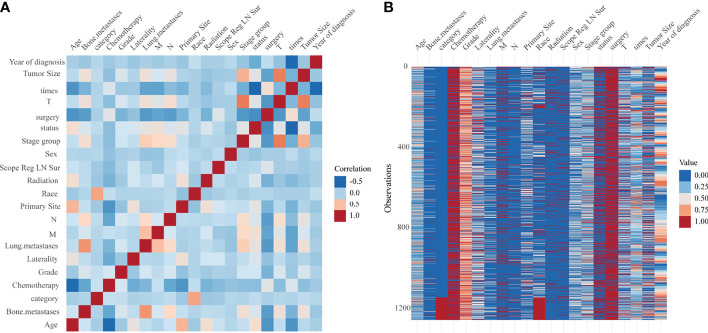
**(A)** Heat map of the correlation between each factor. **(B)** Heat map of the frequency in each factor.

The heat map in **Figure 2A** showed the correlation between each parameter, and that in **Figure 2B** showed the frequency in each parameter. In **Figure 2A**, we could find moderate correlations for tumor size with T and stage group, race with category, and bone metastasis versus lung metastasis. In **Figure 2B**, the frequencies of each parameter were shown, and the data distribution could be observed visually from the colors (**Figure 2**).

### Univariate Cox regression

**Figure 3:**
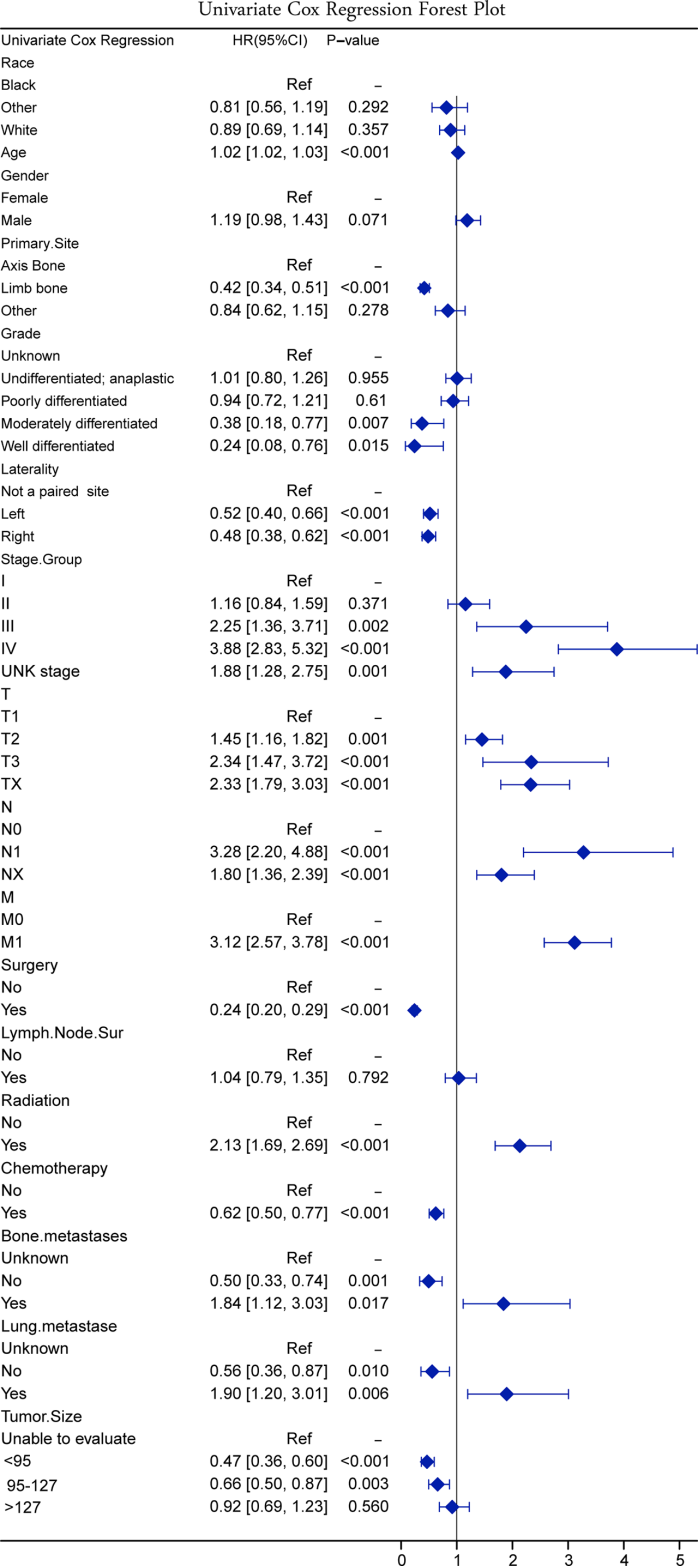
Forest plot on univariate Cox regression.

According to the results of the univariate Cox regression, the forest plot was drawn (**Figure 3**). According to the results of the univariate Cox regression forest plot, 14 variables (*p* < 0.5) were screened by univariate Cox regression, namely, age, primary site, grade, laterality, stage group, T stage, N stage, M stage, surgery, radiation, chemotherapy, bone metastases, lung metastases, and tumor size.

### Full subset regression

**Figure 4:**
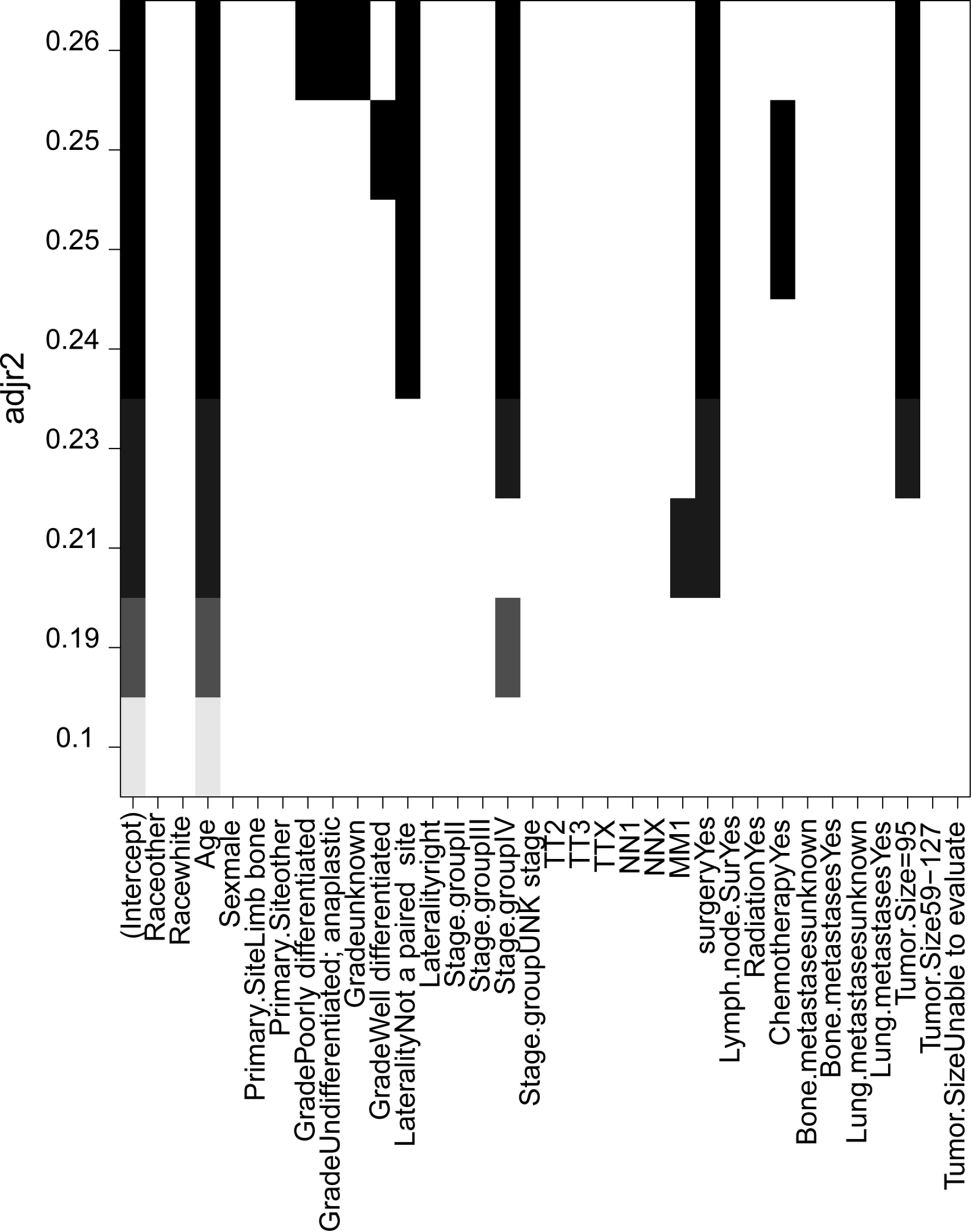
The combination of variables were determined with the adjustment *R*² as criterion in the full subset regression.

The full subset regression was performed using the R packages’ (leaps) regsubsets function to find the best combination according to the optimal subset regression model evaluation criteria, through adjusting the Marlowe’s CP value to minimum, *R* ^2^ value to maximum, and Bayesian information criterion to minimum. The combination of variables was determined with the adjustment *R* ^2^ as criterion. Optimal full subset regression selected eight variables (age, grade, laterality, stage group, M stage, surgery, chemotherapy, and tumor size) (**Figure 4**).

### LASSO regression and cross-validation

**Figure 5:**
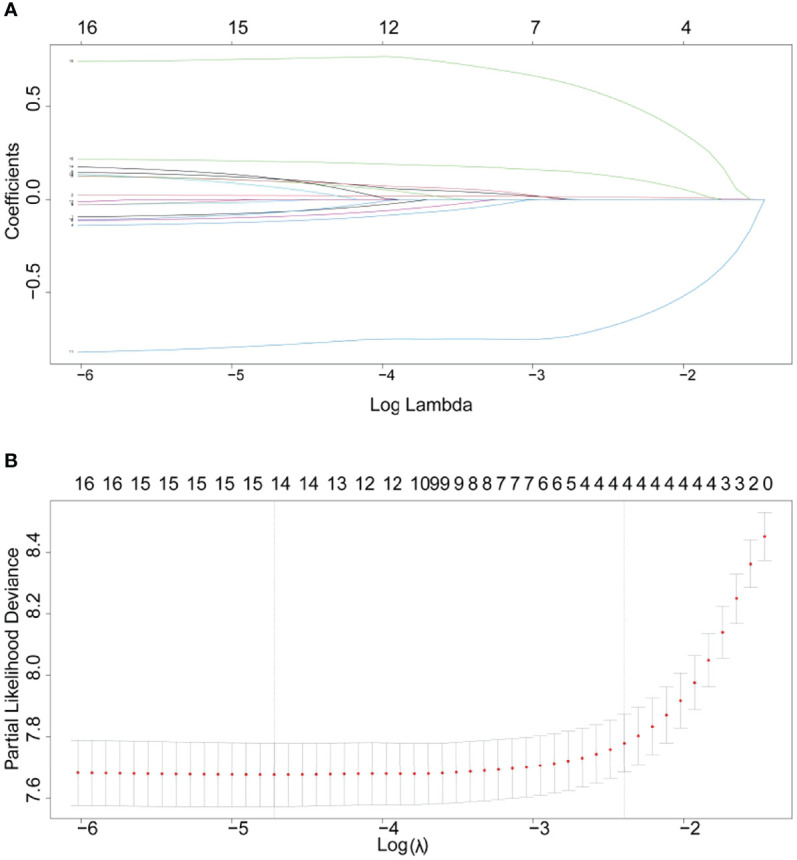
**(A)** LASSO coefficient profile. **(B)** Cross-validation for tuning parameter selection in the LASSO model.

LASSO introduced the variable *λ* to find the most appropriate model. As *λ* increased, the regression coefficient *β* of each variable decreased, and some became zero, indicating that the variable contributed little to the model and could be eliminated. *λ* value determined which variables optimized the model, and the best *λ* value could be found using cross-validation (**Figure 5A**).

**Figure 5B** showed the Partial-likelihood deviance curve with Log(*λ*). The value (*λ*.min and *λ*.1se) was used to choose the good performance model in a minimum number of independent variables. Four combinations of variables (age, M, surgery, and lung metastases) were chosen *via* the LASSO regression.

### Multivariate Cox regression to determine the final model variables

A total of 14 variables were screened by univariate Cox regression. Based on adjusted *R*² maxima, eight variables (age, grade, laterality, stage group, stage M, surgery, chemotherapy, and tumor size) were screened by optimal subset regression (OSR). LASSO regression and cross-validation using a tuning factor (λ.1se) built an excellent model with a minimum number of four independent variables (age, M stage, surgery, and lung metastases).

The combinations of variables screened by each of the three methods were analyzed in a multivariate Cox model, and the final models of the three methods were determined using stepwise backward regression with minimum AIC values. After stepwise backward regression, eight variables were included in the univariate Cox (age, grade, laterality, M, surgery, bone metastases, lung metastases, and tumor size). Six variables were included in the optimal subset regression (age, grade, laterality, M stage, surgery, and tumor size). Four variables were included in LASSO regression (age, M stage, surgery, and lung metastases).

**Figure 6:**
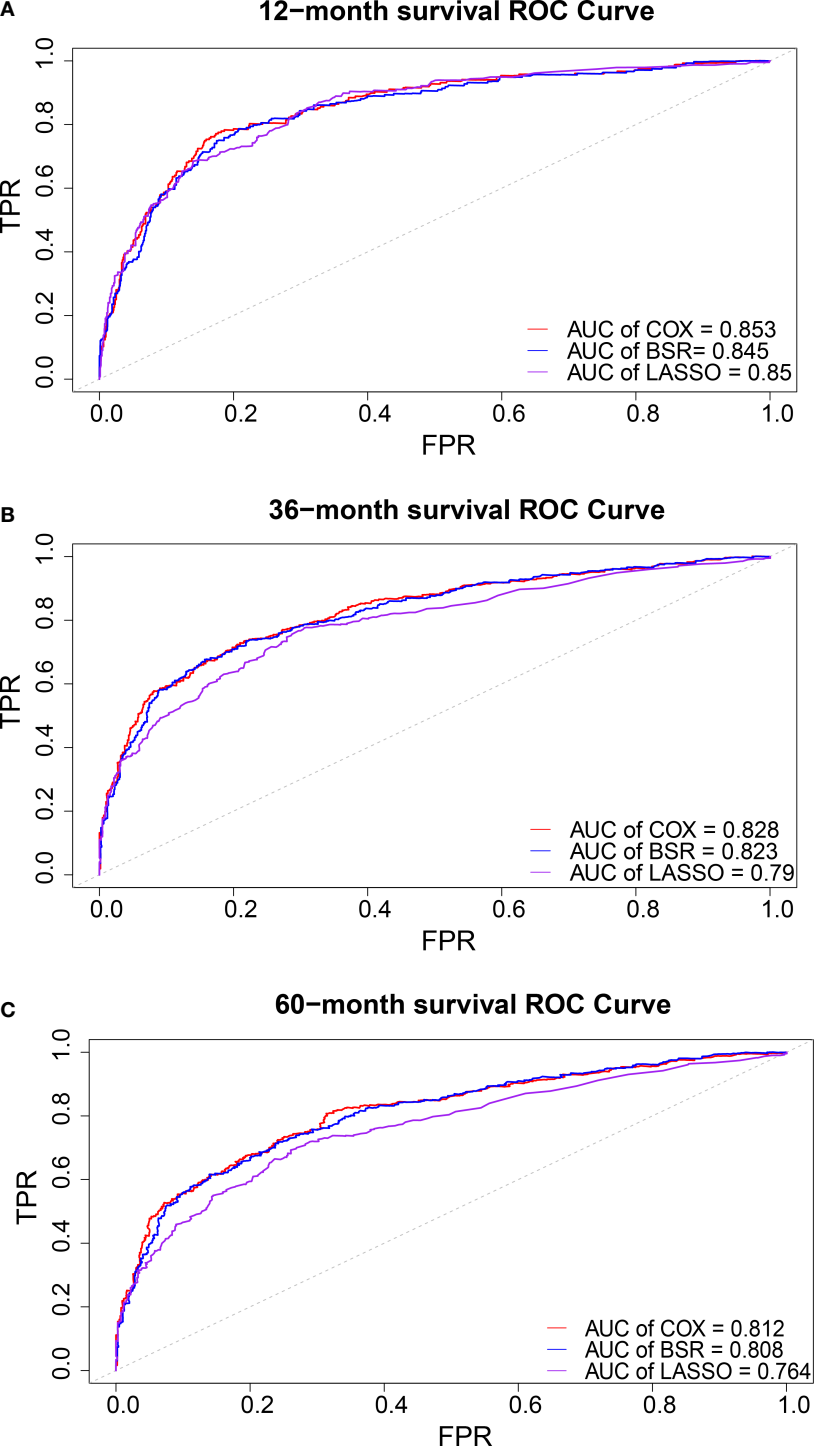
ROC cure in 1-**(A)**, 3-**(B)**, and 5- **(C)** years overall survival.

The AIC of the three models were 5,552.849 in univariate Cox, 5,570.204 in OSR and 5,611.193 in LASSO regression. ROC curves in three models were drawn at 1-year, 3-year, and 5-year survival. The models were evaluated by AUC values (**Figure 6**). The model constructed by univariate Cox was optimal with the largest AUC and the smallest AIC.

### Survival analysis

**Figure 7:**
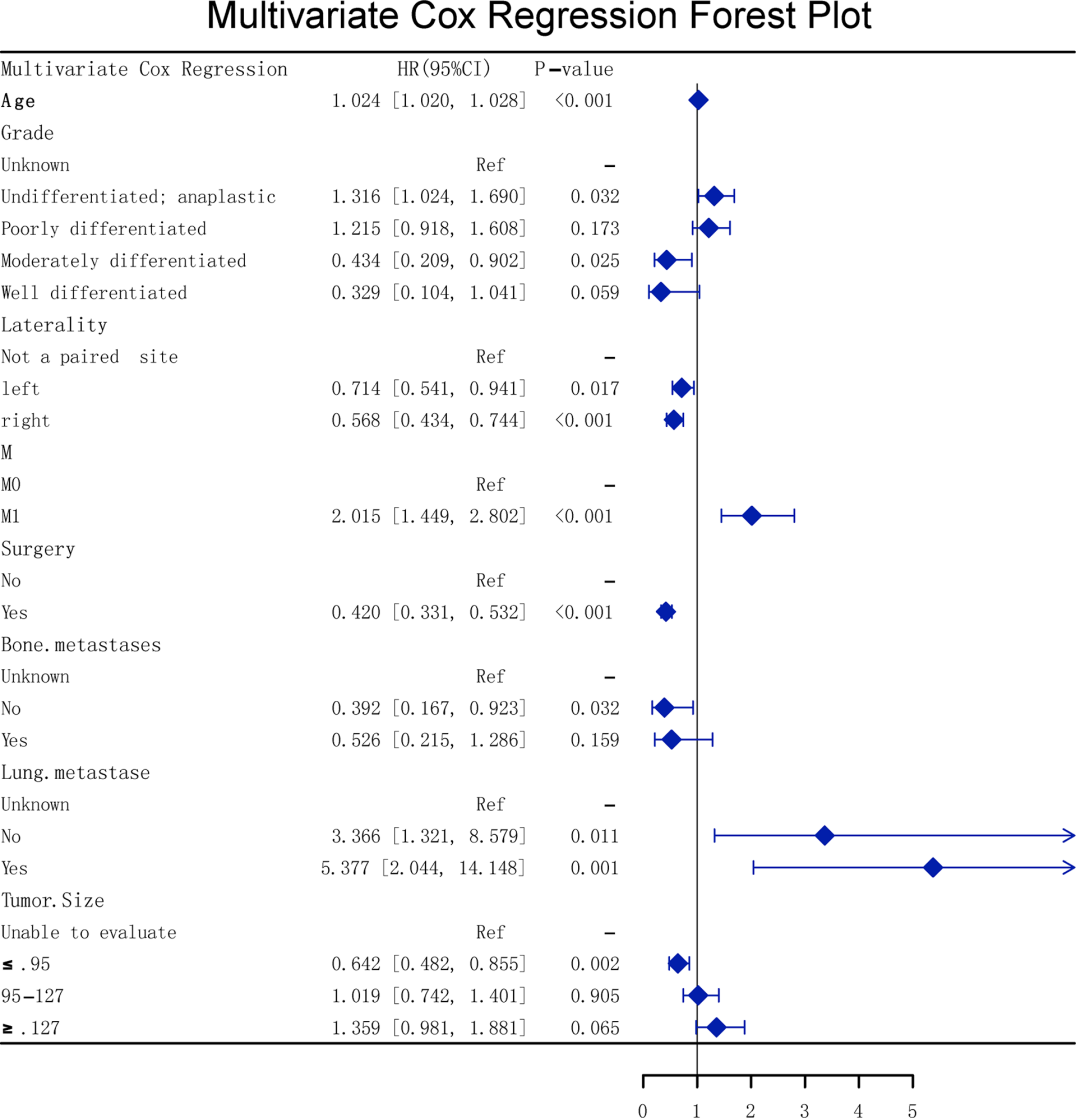
Multivariate Cox forest plot.

The multivariate Cox forest plot showed that eight univariate Cox parameters were independent risk factors (*p* < 0.05, **Figure 7**).

**Figure 8:**
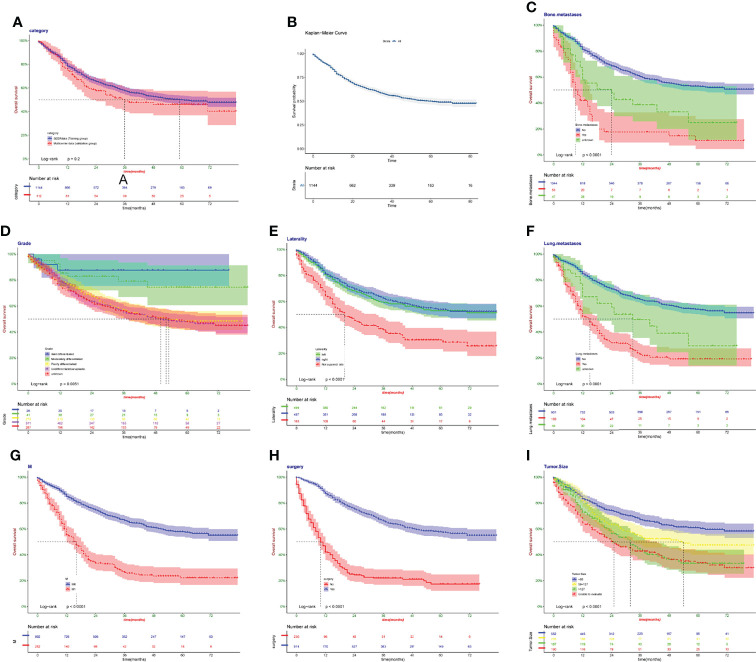
Kaplan–Meier survival curves in the training cohort. **(A)** The SEER data and the real Chinese multicenter data. **(B)** Patients in SEER data. **(C)** Bone metastases. **(D)** Grade. **(E)** Laterality. **(F)** Lung metastases. **(G)** M. **(H)** Surgery. **(I)** Tumor size.

Kaplan–Meier survival curves revealed that there was no significant difference in patient survival between the SEER data and the real Chinese multicenter data (*p* > 0.05, **Figure 8A**). Kaplan–Meier survival curves about all patients are presented in **Figure 8B**. Bone metastases were at higher risk than no bone metastases (*p* < 0.05, **Figure 8C**). Well-differentiated grade patients held longer survival (*p* > 0.05, **Figure 8D**). Kaplan–Meier survival curves almost overlapped in left and right laterality, showing no difference (*p* > 0.05, **Figure 8E**). Lung metastases were at higher risk than no bone metastases (*p* < 0.05, **Figure 8F**). M1 showed a lower survival rate than M0 (*p* < 0.05, **Figure 8G**). Patients with surgery showed higher survival rate than no surgery (*p* < 0.05, **Figure 8H**). The larger the tumor size of patients was, the shorter was their survival (*p* < 0.05, **Figure 8I**). The consistent results were proved in the validation cohort (**Figure S2**).

### Prediction model development

**Figure 9:**
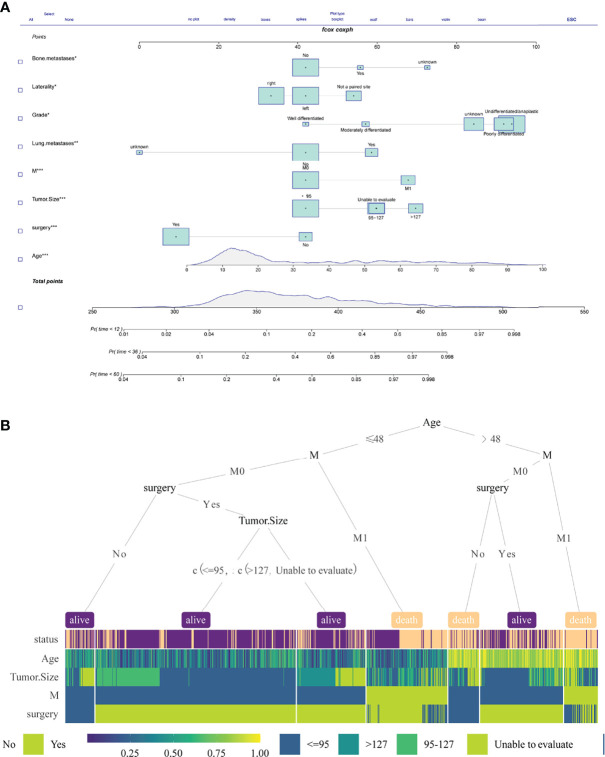
**(A)** A nomogram was constructed using multivariate Cox variables. **(B)** The decision tree of multivariate Cox variables.

A nomogram is a method that allows quantification and visualization of Cox regression (16). The nomogram is evaluated by two methods: (1) Each variable is listed, and each sub-variable is quantified into a specific score. The cumulative scores of all variables are matched to the outcome scale to obtain predicted probabilities. (2) Web calculators or dynamic line graphs are developed to input specific variables and calculate the probability of an event. In this study, we constructed the nomogram using multivariate Cox variables (**Figure 9A**). Moreover, an online web calculator (https://dr-lee.shinyapps.io/osteosarcoma//) was designed to facilitate the user. A decision tree model was also provided as a supplement for the prediction model (**Figure 9B**).

### Calibration chart and external receiver operating characteristic curve

**Figure 10:**
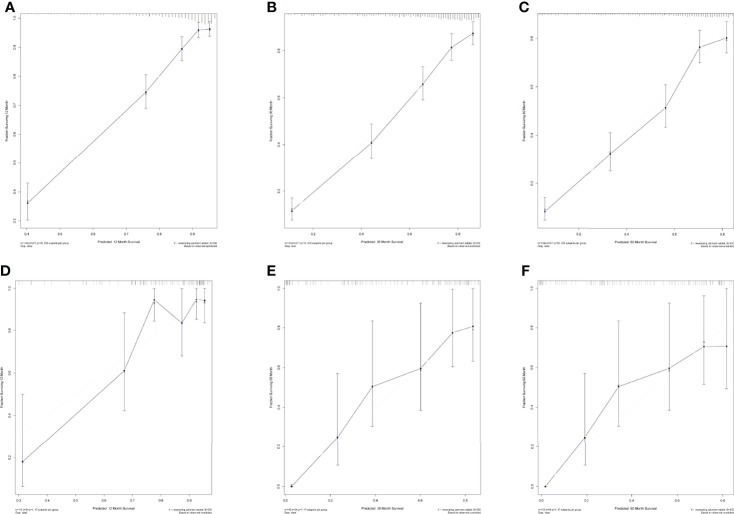
**(A–C)** Internal calibration diagram in 1 year, 3 years, and 5 years. **(D–F)** External calibration diagram in 1 year, 3 years, and 5 years.

The calibration chart was an assessment of how close the estimated risk of the line plot was to the actual risk. SEER data were applied for internal validation, and multicenter data were applied for external validation. The internal validation results (**Figures 10A**–**C**) and external validation results (**Figures 10D**–**F**) showed that the predicting outcomes were consistent with the actual outcome and the prediction model was well preformed in 1, 3, and 5 years. The ROC curves of the model were plotted in multicenter data. It proved the excellent predictive ability in 1, 3, and 5 years (AUC > 0.8, respectively) in **Figure S3**.

### Risk score visualization and decision curve analysis

**Figure 11:**
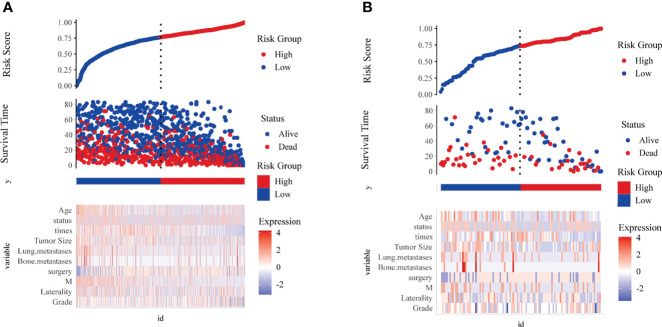
Risk score visualization. The scatter plot of risk score, the scatter plot of survival time and survival status for high and low risk, and the heat map of expression of key risk factors in the training group **(A)** and validation group **(B)**.

The risk score plots were used to visualize Cox survival risk models. **Figure 11** could illustrate the risk factors heat map, the scatter plot of patients’ status, survival time, and high/low risk group, in the training and validation groups, respectively.

**Figure 12:**
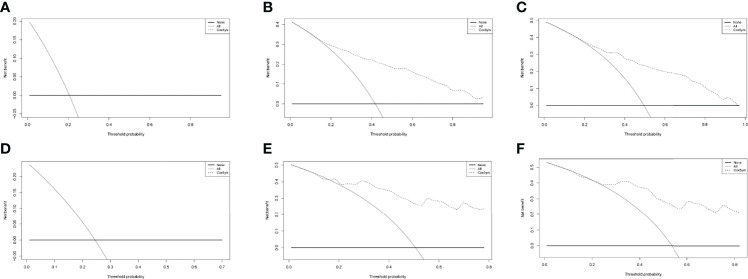
The DCA curves of the nomograms comparison for 1 year **(A)**, 3 year **(B)**, 5 year **(C)** in the training group, and for 1 year **(D)**, 3 year **(E)**, 5 year **(F)** in the validation group, respectively.

As in **Figure 12**, in both the training and validation groups, there was no significant benefit for 1-year patients. In 3 years and 5 years, it was clear that the dashed line received a higher net benefit than the 1 year in both. Considering that osteosarcoma patients do not have a high mortality rate within 1 year of diagnosis, the prediction model developed in this study proved to have excellent clinical utility.

## Discussion

Since the mid-1980s, with the standardization of treatment and the use of adjuvant chemotherapy, the 5-year survival rate for patients with osteosarcoma has arrived to approximately 65% (17). No statistically significant differences were found between osteosarcoma patients from the United States and China, except in the ethnic distribution and the proportion of chemotherapy use (**Table 1**). All races were categorized as white, black, and other in SEER data, with other including Chinese. However, race was excluded as a predictor in three models (univariate Cox, full subset regression, and LASSO). In clinical practice, chemotherapy became a routine treatment option for metastases, especially concomitant lymphatic or vascular micrometastases (18). The proportion of chemotherapy in China was 87.5%, higher than that of the SEER database (**Table 1**). This might be related to the clinicians’ preference and financial cause. Chemotherapy was a protective factor for patients with osteosarcoma in the univariate Cox results (**Figure 3**). However, the overall survival time from the multicenter data was not significantly different from the SEER database (**Figure 8A**). Proper indications for chemotherapy needed future research. This study had limitations since a retrospective study had bias in multicenter data collection, resulting in a higher proportion of chemotherapy.

In 2020, approximately 3,600 new cases with bone tumors and approximately 1,720 patients would die from the malignancy in the United States (19). Osteosarcoma with metastases clinically detected at initial presentation were approximately 20% of all osteosarcoma patients (17, 20). Approximately 30% of patients developed lung metastases within 1 year after diagnosis (21). Early detection of metastases could improve prognosis. Lung metastases had a strong correlation with bone metastases (M stage) and lymphatic metastases (N stage) (**Figure 2**). Bone metastases had a strong correlation with N. The mechanism of lymphatic metastasis from osteosarcoma has not been clear (22). Some studies found that osteosarcoma metastases disrupted the cortex, and the metastatic route might be through the lymphatic vessels of the synovial membrane and bursa (23). The incidence of lymphatic metastasis in patients with osteosarcoma did not exceed 5% in both SEER data and multicenter data, while lymphatic metastasis was a risk factor for patients with osteosarcoma in the univariate Cox results (**Figure 3**). Thus, we suggested that oncologists could not ignore the examination on lymph nodes. A nomogram has been constructed to predict distal metastases from osteosarcoma, which can be used as a method to screen for people at high risk of developing metastases (12). The first peak of mobility occurred at the age of 10–14 years, coinciding with pubertal growth (24, 25). Older patients may be less tolerant to treatment, and had a poorer prognosis. Osteosarcoma in an axial location showed poorer survival, and it was more difficult to completely resect focus due to location. Similarly, the larger tumor volume had poorer prognosis due to difficulty in complete resection, which was similar to previous studies (26).

AJCC (8) and Enneking (9) staging systems can only vaguely assess the clinical risk of osteosarcoma based on initial clinical features to help make treatment decision. Clinical prediction models are widely used today as tools for predicting the occurrence of specific events and estimating medical prognosis, especially in clinical oncology. Clinical prediction models generate probabilities of individual clinical events by integrating different predictor and decision variables, and their visualization and quantification advantages are also of great practical value in clinical practice (27). Most prediction models are developed based on logistic regression and Cox regression models. However, the full model equation remains difficult. In our study, three models (univariate Cox, full subset regression, and LASSO) were performed in the SEER database. Univariate Cox model and eight predicting factors (age, grade, laterality, stage M, surgery, bone metastases, lung metastases, and tumor size) were selected based on the minimum AIC and maximum AUC value. The model was further externally validated and evaluated for its clinical utility with data from four medical centers in China. ROC curves revealed good predictive ability (AUC > 0.8 in both internal and external validation, **Figure 6**).

A nomogram and web calculators were applied and visualized. Decision trees were provided as prediction model aids. A major advantage of the web calculator is that, compared to a rating scale or approximations calculated by the nomogram, the full model equation can be embedded in a backend web page, is more accurate in its calculation, and is more convenient to use. Web calculators can provide user-friendly graphical interfaces for complex mathematical models reducing the learning cost for users in today’s world of smartphones and mobile networks. The nomogram is an effective quantitative method to assess risk and benefit and is widely used in clinical decision-making in a variety of diseases (28). In previous studies, several nomograms have been developed and validated to predict specific survival and overall survival in chondrosarcoma (29, 30).

In this study, a clinical prediction model to predict the overall survival of patients with osteosarcoma was developed to provide an objective reference for clinicians when making medical decisions. In clinical practice, lacking large-scale prognosis statistics for osteosarcoma patients in China, we chose the SEER database to develop the prediction model, and collected patient data from four medical centers in China to verify the feasibility of the model. In terms of clinical utility, the risk factor plots showed good stratification in both cohorts, effectively differentiating between high- and low-risk patient populations (**Figure 10**). The DCA displayed that both cohorts had better patient benefit from medical interventions in 3 years and 5 years. The 1-year model did not have a great net benefit, which may be related to the low mortality within 1 year (31).

Despite our efforts to refine the clinical prediction model, some limitations remained. (1) Training data (SEER database) to develop the prediction model were from North American patients, while the multicenter external data of China were tested for the model’s predictive power. (2) The model was based on retrospective data and inevitably had inherent biases. (3) Previous studies showed that metastasis of osteosarcoma was associated with genes, metastatic mechanisms, proteins, and RNAs (32, 33). Since the SEER database did not contain relevant information, there was still room to improve the predictive power of the model.

## Conclusions

In this study, based on the SEER database and data of osteosarcoma patients from 4 different regional medical centers in China, the model with the highest predictive ability was selected by three methods of screening model predictors, and the model was visualized for predicting the overall survival of osteosarcoma patients using three methods: nomogram, web calculator, and decision tree. The model was shown to have very good predictive power and consistency by both calibration plots and ROC curves. DCA demonstrated that the predictive model could provide greater benefit to patients. External validation results show that it still has predictive power and clinical use outside of North America.

## Data availability statement

The data analyzed in this study is subject to the following licenses/restrictions: SEER database within the article is public data set. The clinical information data of China’s multicenter, analyzed during the current study are not publicly available for patient privacy purposes, but are available from the corresponding author upon reasonable request. Requests to access these datasets should be directed to CY, chengliangyin@163.com.

## Ethics statement

This study was exempted from Institutional Review Board approval, in view of the SEER’s use of unidentifiable patient information. Due to the strict register-based nature of thestudy, informed consent was waived. The study of multicenter data was approved by the ethics review committee of the Second Affiliated Hospital of Jilin University, the Second Affiliated Hospital of Dalian Medical University, Liuzhou People’s Hospital, and Xianyang Central Hospital (No. 20210021).

## Author contributions

CLY, WZ, HWP: study conception and design; WLL, GYH: manuscript writing; HTW, RLGW, CX, BW, QL: literature review; all authors: data interpretation and discussion; all authors: final editing and approval of the manuscript in its present form.

## Acknowledgments

We thank all individuals who took part in this research.

## Conflict of interest

Author HW was employed by the company Baidu Inc.

The remaining authors declare that the research was conducted in the absence of any commercial or financial relationships that could be construed as a potential conflict of interest.

## Publisher’s note

All claims expressed in this article are solely those of the authors and do not necessarily represent those of their affiliated organizations, or those of the publisher, the editors and the reviewers. Any product that may be evaluated in this article, or claim that may be made by its manufacturer, is not guaranteed or endorsed by the publisher.

## Supplementary material

Supplementary Figure 1 Flow chart of data collection and analysis

Supplementary Figure 2 Kaplan–Meier survival curves in validation cohort. **(A)** the SEER data and the real Chinese multicenter data. **(B)** patients in multicenter data. **(C)** Bone metastases. **(D)** grade. **(E)** Laterality. **(F)** Lung metastases. **(G)** M. **(H)** surgery. **i** tumor size.

Supplementary Figure 3 External ROC curve in 1 year, 3 years, 5 years.

## Abbreviations

AJCC, The American Joint Committee on Cancer; AIC, Akaike’s Information Criterion; AUC, area under the curve; DCA, decision curve analysis; MSE, mean squared error; ROC, receiver operating characteristic curve; SEER, The Surveillance, Epidemiology, and End Results.
