# Supplementary figures and images for: Interpretable clinical visualization model for prediction of prognosis in osteosarcoma: a large cohort data study
Source: Front Oncol. 2022 Aug 2;12:945362. doi: 10.3389/fonc.2022.945362 (PMC9394445; doi:10.3389/fonc.2022.945362)

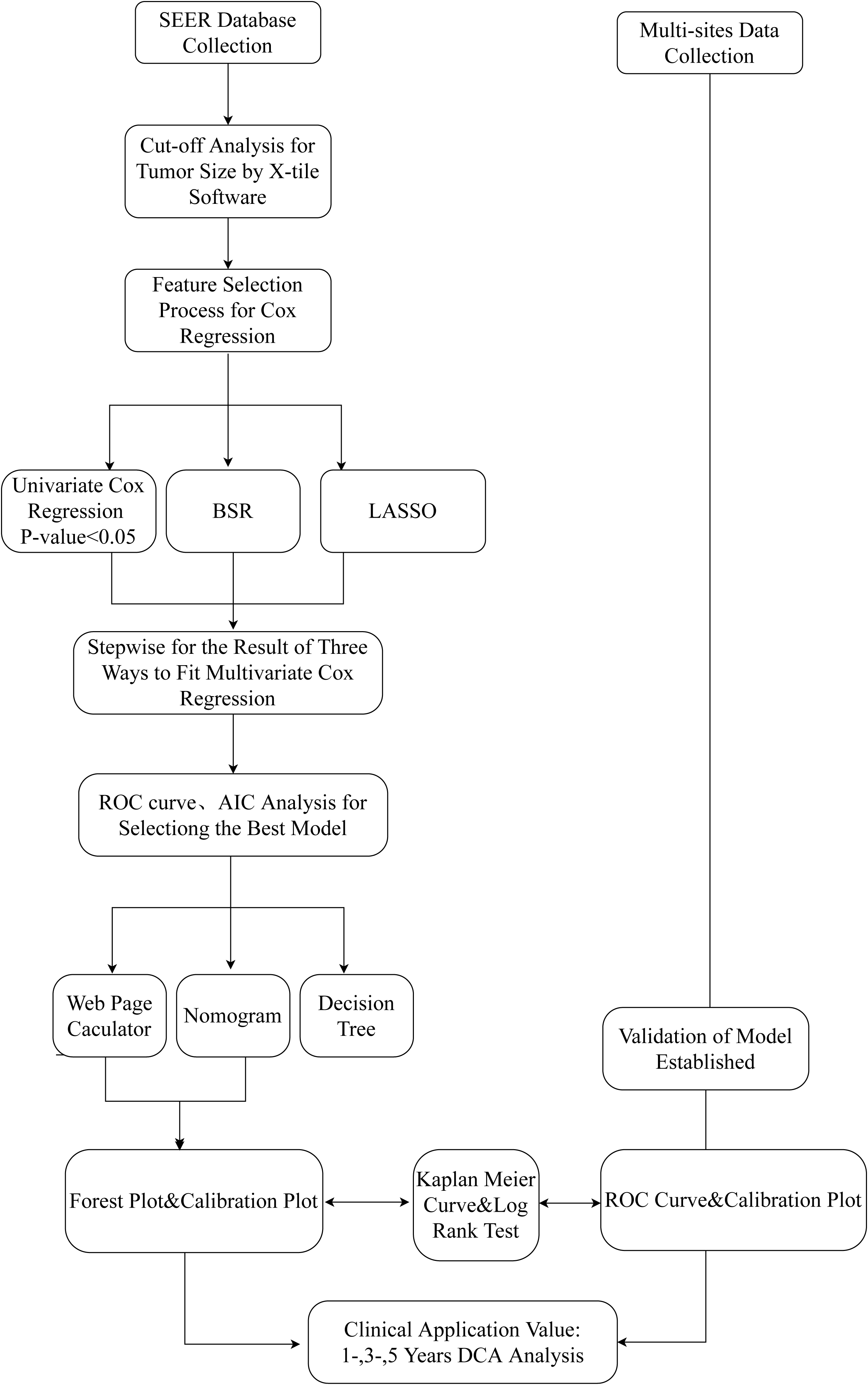

Supplement: Supplementary Figure 1 — Flow chart of data collection and analysis [file Image_1.jpeg]

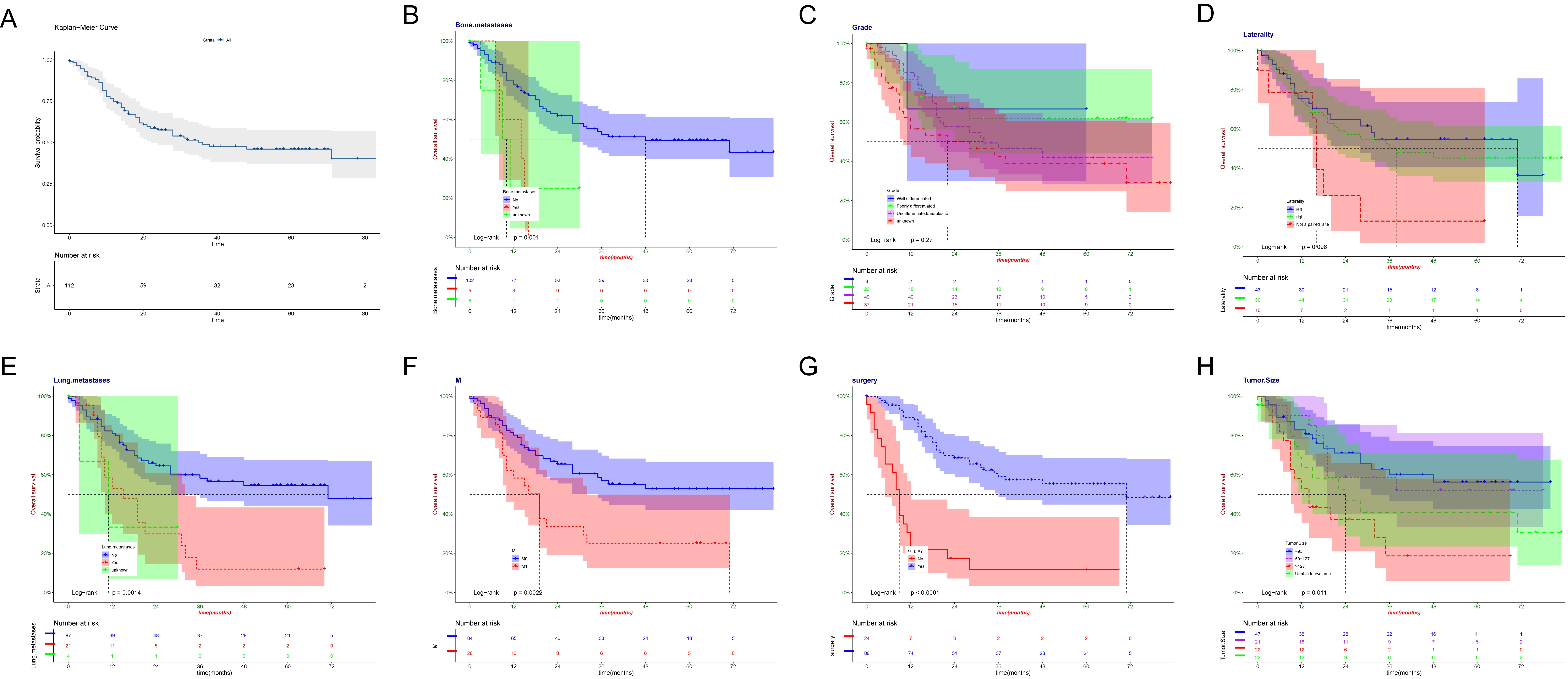

Supplement: Supplementary Figure 2 — Kaplan–Meier survival curves in validation cohort. (A) the SEER data and the real Chinese multicenter data. (B) patients in multicenter data. (C) Bone metastases. (D) grade. (E) Laterality. (F) Lung metastases. (G) M. (H) surgery. i tumor size. [file Image_2.jpeg]

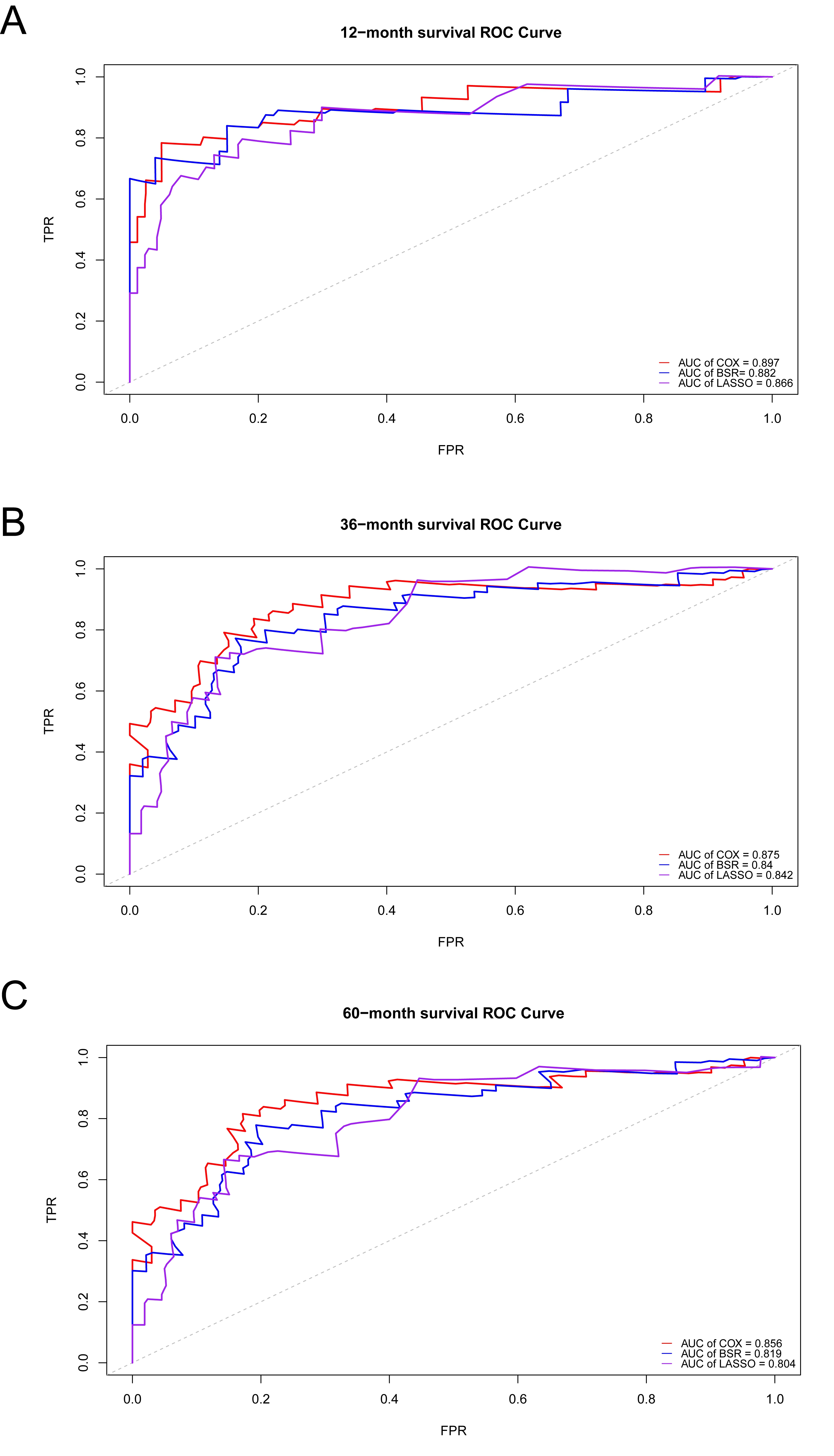

Supplement: Supplementary Figure 3 — External ROC curve in 1 year, 3 years, 5 years. [file Image_3.jpeg]
